# Enhancing Robotic Grasping Detection Using Visual–Tactile Fusion Perception

**DOI:** 10.3390/s26020724

**Published:** 2026-01-21

**Authors:** Dongyuan Zheng, Yahong Chen

**Affiliations:** 1 School of Artificial Intelligence, Lishui University, Lishui 323000, China; zhengdongyuan@mails.ccnu.edu.cn; 2 School of Computer Science, Central China Normal University, Wuhan 430079, China

**Keywords:** robotic grasping, visual–tactile fusion, grasp stability, tactile sensing, deep learning

## Abstract

With the advancement of tactile sensors, researchers increasingly integrate tactile perception into robotics, but only for tasks such as object reconstruction, classification, recognition, and grasp state assessment. In this paper, we rethink the relationship between visual and tactile perception and propose a novel robotic grasping detection method based on visual–tactile perception. Initially, we construct a visual–tactile dataset containing the grasp stability for each potential grasping position. Next, we introduce a novel Grasp Stability Prediction Module (GSPM) to generate a grasp stability probability map, providing prior knowledge regarding grasp stability to the grasp detection network for each possible grasp position. Finally, the map is multiplied element-wise with the corresponding colored image and inputted into the grasp detection network. Experimental results demonstrate that our novel visual–tactile fusion method significantly enhances robotic grasping detection accuracy.

## 1. Introduction

Grasping is a profoundly interactive task [1]. Humans engaging in object grasping frequently employ a fusion of sensory inputs, incorporating both visual and tactile sensing rather than relying solely on one modality [2]. Vision instinctively directs the grasp poses, while tactile captures nuanced contact details—both pivotal elements in determining the prospect of a successful grasp. This has prompted a movement in robotics to emulate human capabilities by integrating information from both visual and tactile modalities to improve grasping performance.

However, the incorporation of tactile sensing into robotic grasping faces significant challenges, including issues related to sensor sensitivity, cost constraints, and the complexities of integrating tactile inputs into conventional control schemes. Consequently, the predominant input modalities extensively explored in previous research on robotic grasping primarily revolve around vision and depth [3,4]. Currently, some research endeavors [5,6] have aimed to address this challenge through the implementation of visual–tactile fusion perception.

Many existing methods for visual–tactile fusion perception primarily concentrate on tasks such as evaluating grasp states [7,8], reconstructing objects [9,10], recognizing objects [11,12], etc. However, these methods often limit themselves to straightforward concatenation of visual and tactile information, without delving into a deeper exploration of the intricate relationship between tactile and visual data.

**Figure 1:**
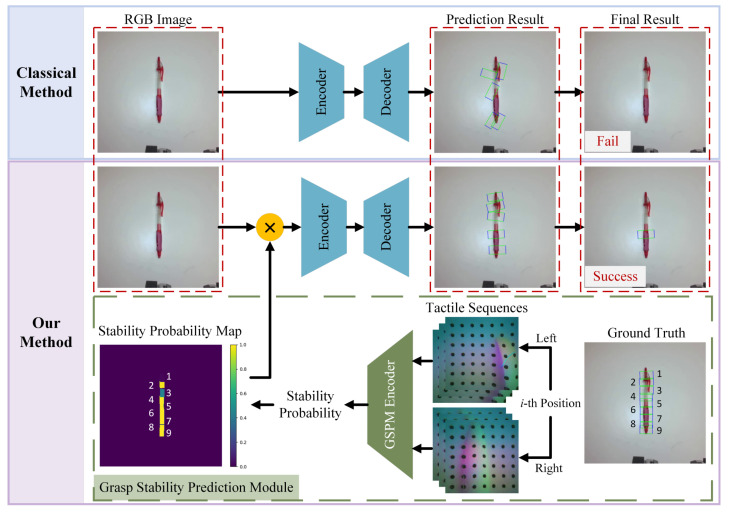
Comparison between our method and classical methods. Our method introduces a Grasp Stability Prediction Module (GSPM) to forecast the stability of potential grasp positions and leverages prior knowledge about grasp stability to guide the grasp detection network, leading to more informed decision-making during grasping compared to classical methods that may not consider such contextual information. **Note: The GSPM is exclusively utilized during the training phase**.

As illustrated in Figure 1, compared with classical grasping detection methods [13,14,15], our method introduces a Grasp Stability Prediction Module (GSPM) to enhance grasping detection accuracy. This module predicts the probability of grasp stability for each potential grasp position of objects using tactile information, thus generating a stability probability map for the grasped object. Subsequently, this map is element-wise multiplied with the corresponding colored image and then fed into the grasping detection network to obtain the final grasping rectangle.

This paper’s main contributions are as follows:

- **Visual–tactile grasp dataset**. Compared to existing grasping datasets [16,17,18,19,20,21], our collected dataset not includes only visual information but also tactile data for each potential grasping position of the grasped objects.
- **Grasp Stability Prediction Module (GSPM)**. Humans start developing their grasping ability from childhood, gradually acquiring the skill to effortlessly grasp objects. Inspired by this developmental process, we introduce a GSPM to emulate human prior knowledge of grasping strategies. The GSPM pre-informs the grasping detection network about stable positions, prompting the network to prioritize attention to these positions rather than spreading focus across all potential grasping positions.
- **Visual–tactile fusion**. In this paper, our proposed visual–tactile fusion method mimics human behavior. Tactile perception evaluates the grasping stability of each potential grasping position, while visual perception utilizes prior knowledge from tactile perception to determine the optimal grasping position. This sequential processing flow enables the acquisition of an optimal grasping strategy that leverages both visual and tactile information effectively. Unlike prior visuo-tactile works that focus on task-specific control or grasp refinement, this work aims to establish a general grasp detection schema that can flexibly accommodate tactile information.

## 2. Related Work

### 2.1. Robotic Grasping Detection

The evolution of building grasping detection datasets reflects a progression from rudimentary approximations of grasping rectangles to more precise identification methods. This advancement has been driven by the rapid development of data-driven techniques, resulting in significant improvements in robotic grasping detection. For instance, Zhai et al. [22] addressed the need for a balance between real-time performance and accuracy in grasping detection networks by introducing a novel network architecture based on grasp keypoints. Meanwhile, Wu et al. [23] tackled the challenge of achieving robust robotic grasping in cluttered environments through an information-theoretic exploration approach, enhancing grasp estimation quality amidst complexity. Huang et al. [24] targeted the issue of six-DoF grasp pose detection with a novel neural network model aimed at increasing grasp success rates. Additionally, Cheng et al. [25] developed a deep grasp detector tailored for robots equipped with parallel grippers, enabling the automated grasping of diverse, previously unseen objects.

The aforementioned methods rely on visual information for robotic tasks, overlooking the crucial tactile sensing modality, whereas our method integrates tactile perception to enhance the accuracy of grasp detection.

### 2.2. Visual–Tactile Fusion in Robotics

Tactile perception plays a critical role in robotic functionality, enabling robots to perform complex and delicate tasks. With the rapid advancement of tactile sensors such as Gelsight [26], Digit [27], the TacTip family [28], and TIRgel [29], which can better learn the geometry and texture of the objects with varying object sizes and curvatures, researchers are increasingly integrating tactile perception into robotics. For instance, Zhang et al. [30] introduced a deep visual–tactile predictive model capable of learning servoing tasks iteratively from raw sensor data. This model empowers robots to proficiently unfold back-opening hospital gowns and execute upper-body dressing maneuvers. Furthermore, Zhang et al. [31] proposed an attention-guided cross-modality fusion architecture, facilitating the comprehensive integration of visual and tactile features. To enhance a robot’s object manipulation capabilities, Liu et al. [32] developed a tactile-enhanced 6D pose tracking system capable of tracking previously unseen objects held in hand. Additionally, Dong et al. [33] presented a novel lifelong visual–tactile learning model specifically designed for continuous robotic visual–tactile perception tasks, effectively exploring latent correlations within both intra-modality and cross-modality aspects.

However, existing methods often overlook the intrinsic relationships across different modalities and the shared characteristics among tasks within each modality. Our method rethinks and explores the relationship between visual and tactile perception to address this limitation.

## 3. Visual–Tactile Data Collection

### 3.1. Data Collection Platform

To advance our research objectives, we establish a comprehensive visual–tactile data collection platform. This setup comprises pivotal components: a six-axis robot arm (JAKA MiniCobo, JAKA Robotics Co., Ltd., Shanghai, China), a two-jaw parallel gripper (PGE-50-26, DH-Robotics Technology Co., Ltd., Shenzhen, China), an RGB-D camera (Intel RealSense D435i, RealSense, Inc., San Francisco, USA), a Linux-based computer operating on Ubuntu 20.04.6 LTS, and two tactile sensors (GelSight Mini, GelSight, Inc., Waltham, USA). The two-jaw parallel gripper is attached to the end of the robot arm to facilitate grasping operations. For tactile data acquisition, we replace the gripper fingers with GelSight Mini sensors, renowned for their capacity to capture intricate surface details. Furthermore, the Intel RealSense camera mounted on the robotic arm captures colored images of grasped objects for visual data acquisition. These sensors seamlessly transmit captured images to the computer for subsequent analysis. This comprehensive setup empowers us to amass a wealth of visual and tactile data, significantly bolstering our research endeavors in robotic perception and manipulation.

### 3.2. Data Collection Strategy

In order to attain prior knowledge of grasping comparable to human capability, it is imperative to gather tactile data pertaining to every potential grasping position. Initially, we annotate all feasible grasping rectangles for each object using the method proposed by Wang et al. [34]. Subsequently, we systematically acquire tactile data corresponding to each labeled grasping rectangle.

**Figure 2:**
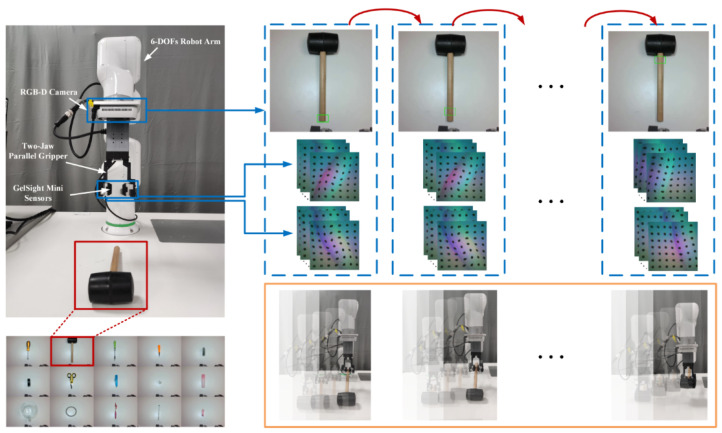
Visual–tactile data collection. Each object presents multiple potential grasping positions, each characterized by varying levels of grasp stability. For instance, consider grasping a hammer: the proximity to the head position typically offers greater grasp stability compared to other positions. This discrepancy arises due to factors such as object geometry, weight distribution, and surface texture. As a result, we systematically collect tactile data during the grasping process at each possible grasp position. Subsequently, we label this data based on whether the grasp is successful or unsuccessful.

As shown in Figure 2, our methodology entails initiating tactile data collection from the first grasping rectangle associated with its label. This requires pre-programming the robot arm to execute the grasping process, comprising approaching, grasping, and lifting phases, with a total duration in less than 1 s. Consequently, the tactile sensor captures left/right tactile data. After preprocessing tactile video sequences, each video contains 36 frames with a frame frequency of 60 Hz. We define a stable grasping state as one where the object does not slip or drop during the lifting phase, labeled as ‘1’, and unstable grasping state as the opposite, labeled as ‘0’. Subsequently, we assign either ‘1’ for stable or ‘0’ for unstable to each grasping video based on this criterion.

In summary, we labeled each of the possible grasping positions for a total of 15 daily grasped objects. As depicted in Table 1, these objects exhibit variations in geometry, size, and material properties, such as rigid metallic tools, plastic containers, and glass objects. The data collection pipeline resulted in the collection of 126 grasping videos, corresponding to the labeled grasping positions. Each video initially had a resolution of 640 × 480 pixels.

## 4. Methodology

**Figure 3:**
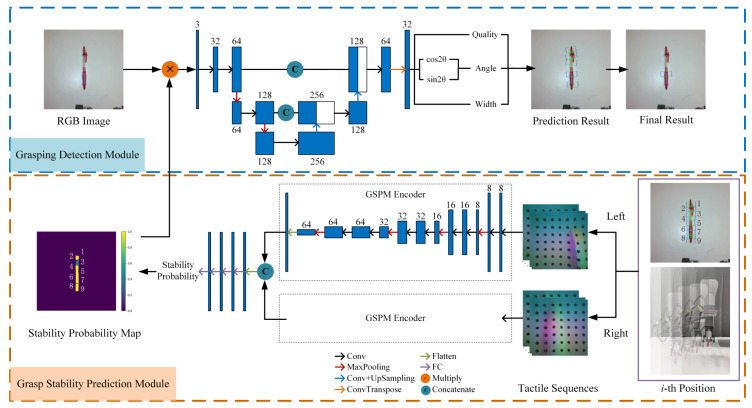
Overview of network framework. The Grasp Stability Prediction Module (GSPM) is exclusively utilized during the training phase and is not employed during the prediction phase. This process closely mirrors the human grasping process: just as humans utilize prior knowledge to determine the optimal grasping position before executing the grasp, our model utilizes the grasp stability predictions generated by the GSPM during training to inform its decision-making process. Once trained, the model relies solely on visual information to predict the optimal grasping position, akin to how humans make real-time grasping decisions based on sensory inputs. This approach enables our model to emulate the cognitive aspects of human grasping.

As shown in Figure 3, our proposed method comprises two primary components: a Grasp Stability Prediction Module (GSPM) and a Grasping Detection Module (GDM). The GSPM primarily forecasts the grasp stability value based on tactile information at each potential grasping position of the object. It analyzes tactile data to assess the likelihood of a stable grasp at various locations on the object and generates a grasp stability map. Conversely, the GDM integrates both a visual and grasp stability map (tactile perception) to predict the grasping rectangle. Leveraging both visual and tactile information, the GDM synthesizes a comprehensive understanding of the object’s characteristics and environmental conditions. Below, we provide a detailed description of each component, outlining their functionalities and contributions to the overall grasping detection process.

### 4.1. Grasp Stability Prediction Module

The GSPM receives tactile video sequences captured from both the left $I_{left}$ and right sides $I_{right}$ of the GelSight tactile sensor, with each frame resized to 224 × 224 pixels. The GSPM’s encoder (see Figure 3), denoted as $E_{GSPM}$, combines convolutional and maxpooling layers to extract features from frames of the input tactile video sequences, utilizing a convolution kernel with 3 × 3. The output feature maps $F_{left}$ and $F_{right}$ from the GSPM’s encoder can be represented as

(1) $$\begin{array}{cc} \; & F_{left}=E_{GSPM}\left(I_{left}\right) \end{array}$$

(2) $$\begin{array}{cc} \; & F_{right}=E_{GSPM}\left(I_{right}\right) \end{array}$$

The output feature maps are concatenated and subsequently pass through a series of fully-connected layers after the Flatten layer. Let $F_{c}$ denote the concatenated feature maps, *Concat* denote concatenation operation, and *Flatten* denote flatten operation. The operation of flattening followed by concatenation can be represented as

(3) $$F_{c}=\mathrm{Flatten}\left(\mathrm{Concat}\left(F_{left},F_{right}\right)\right)$$

$F_{c}$ is then processed by a series of fully-connected layers, and the final fully-connected layer employs the Sigmoid function to compute the stability probability of the object grasping position. Let ${\mathrm{FC}}_{n}$ represent the *n^th^* fully connected layer with weight matrix $W_{n}$ and bias vector $b_{n}$. The computation of the stability probability $P_{s}$ can be represented as

(4) $$P_{s}=\sigma \left({\mathrm{FC}}_{n}\left(\ldots {\mathrm{FC}}_{1}\left(W_{1}\cdot F_{c}+b_{1}\right)\ldots \right)+b_{n}\right))$$

where σ represents the Sigmoid function, and *n* is equal to 4.

### 4.2. Stability Probability Map

**Figure 4:**
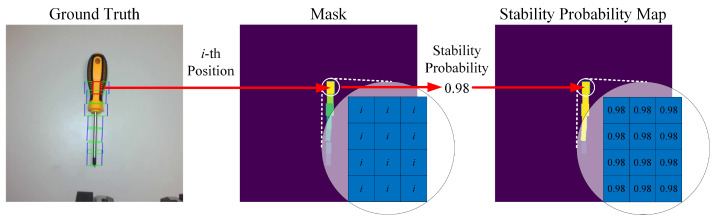
Generation process of stability probability map. The red rectangle in the ground truth indicates that the center third of each grasping rectangle serves as an image mask corresponding to the position of the gripper’s center.

The stability probability map is capable of providing prior knowledge regarding grasp stability to the grasp detection network for each possible grasp position, as shown in Figure 4. We use grasping rectangles to represent the object’s grasping position, and the center third of each grasping rectangle is used as an image mask that corresponds to the position of the gripper’s center. Specifically, we define the mask M(x,y) as follows:

$$M\left(x,y\right)=\left\{\begin{array}{cc} i, & \mathrm{if}\;\left(x,y\right)\in R_{i}\\ 0, & \mathrm{otherwise} \end{array}\right.$$

where $R_{i}$ denotes the *i*-th grasping rectangle. Within the mask region, pixels are assigned values from 1 through *i* in sequence of the grasping positions, where *i* indicates the number of object grasping positions. The other pixels in the image are set to 0.

As mentioned in Section 4.1, we can obtain the stability probability $P_{s}$ of each grasping position from the GSPM. In the next step, the pixels in the masks are set to the stability probabilities of the grasping positions corresponding to them. Eventually, all the values of the masks in the image are stability probability values, and the values in the rest of the image are 0. We refer to this image as the stability probability map.

$$M^{\prime }\left(x,y\right)=\left\{\begin{array}{cc} P_{s}^{i}, & \mathrm{if}\;\left(x,y\right)\in R_{i}\\ 0, & \mathrm{otherwise} \end{array}\right.$$

### 4.3. Grasping Detection Module

The GDM architecture is based on a U-structured Convolutional Neural Network, as illustrated in Figure 3. The input of the GDM comprises both the RGB image of the grasped object and the predicted grasping stability probability map, both of which are 468 × 468 pixels. These inputs are combined using element-wise multiplication to fuse visual and tactile information.

**Figure 5:**
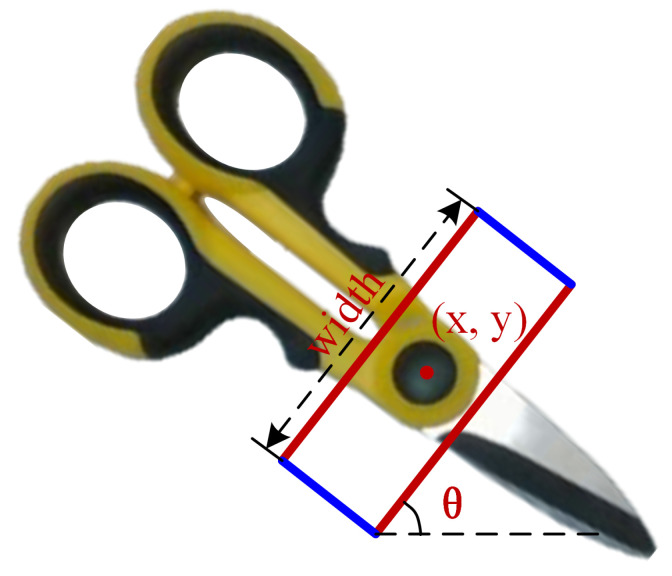
Representation of the grasp rectangle.

As depicted in Figure 5, we employ a two-dimensional image grasping representation, denoted as

(5) $$g_{i}=\left\{x_{c},y_{c},\theta_{i},w,q\right\}$$

where $\left(x_{c},y_{c}\right)$ denotes the center point of the grasping rectangle, and $\theta_{i}$ represents the grasping angle of the robot gripper at the *i*-th position, with an angle range of $\left[-\frac{\pi }{2},\frac{\pi }{2}\right]$. *w* indicates the width of the gripper jaws opening, with a range of $\left[0,w_{\max}\right]$. *q* signifies the grasping quality, with values ranging from 0 to 1. A value close to 1 indicates a high probability of the object being successfully grasped by the robot at that position. To avoid discontinuities at $\theta_{i}=\pm \frac{\pi }{2}$ and facilitate smooth optimization in the angular space, the grasp angle $\theta_{i}$ is commonly encoded using its cosine and sine components with a $2\theta_{i}$ parameterization. Accordingly, the grasp representation at the center pixel of the *i*-th grasp rectangle in a two-dimensional image is defined as

(6) $$g_{i}=\left\{q_{i},\cos2\theta_{i},\sin2\theta_{i},w_{i}\right\}.$$

The GDM output consists of four components: grasping quality, cos2θ, sin2θ, and gripper opening width. The gripper position is determined as the location with the highest grasp quality in the grasp quality image. Once the gripper position is identified, the corresponding angle and width in other images can be extracted. The gripping angle is calculated as

(7) $$\theta =\frac{1}{2}arctan\frac{sin2\theta }{cos2\theta }$$

## 5. Experiments and Results

### 5.1. Implementation and Experimental Setup

We conduct our experiments using Pytorch on an NVIDIA GeForce RTX 4090 server. Distinct experimental parameter settings are employed for the two modules within our framework.

In the GSPM module, we utilize binary cross-entropy as the loss function and apply a sigmoid activation function to predict the probability of the object grasping position stability. We employed the Adam optimizer with a learning rate set to 0.001, without incorporating learning rate decay. During training, the network model underwent 15 epochs, and the weights corresponding to the optimal performance were retained. To obtain a stability probability map for the grasped object, we initially divided tactile video sequences into two equal parts, designated as the training dataset and the test dataset. Subsequently, we trained the GSPM using the training dataset and utilized the trained GSPM to predict stability probability values for each potential grasping position on the test dataset. This process yielded stability probability values for the training dataset. We then interchanged the training and test datasets, repeating the aforementioned steps to acquire the remaining stability probability values. Finally, we utilized these stability probability values to replace the values within the corresponding grasping rectangle boxes.

In the GDM module, we initially conduct data augmentation, including rotation and zoom operations, resulting in a dataset comprising 1500 RGB images along with their corresponding grasping stability probability maps. In our experiments, 10 objects were used for training and 5 unseen objects for testing, which means the training and testing sets were object-disjoint, and 5-fold cross-validation was used to evaluate generalization to unseen objects. We employed the Smooth L1 loss function, also known as the Huber loss function, and utilized the Adam optimizer with a learning rate of 0.001. The GDM underwent training for a total of 100 epochs, after which the optimal weights were saved for further use.

### 5.2. Results and Discussion

To assess the effectiveness of our proposed method, we integrated the Grasp Stability Prediction Module (GSPM) into various grasping detection models, including the widely recognized models GG-CNN [14] and GR-CNN [35] within the domain of 2D planar grasping detection.

We evaluated our proposed GSPM across various grasping detection methods. In these comparative experiments, we adhered to the following evaluation criteria for grasping detection success:

1. Intersection over Union (**IoU**) between predicted grasp rectangles and ground truth, with thresholds set to 0.4, 0.3, and 0.25.
2. Angular (**∆angle**) difference between predicted grasp rectangles and ground truth angles, with thresholds set to 15°, 10°, and 5°.

As depicted in Table 2, we obtained the grasping detection accuracy of different methods and evaluation standards. The grasping detection method with the GSPM (***w*** **GSPM**) indicates the utilization of both visual and tactile information, while ***w*****/*****o*** **GSPM** denotes solely relying on visual information. While easy evaluation standards may indicate a high grasping detection accuracy, they may not adequately reflect real-world robot operation requirements. Notably, as evaluation standards become more stringent, grasping detection accuracy experiences a rapid decline when GSPM is not employed. Conversely, when the GSPM is utilized, grasping detection accuracy consistently remains above 0.8 across various methods. Therefore, when confronted with strict evaluation standards, leveraging both visual and tactile information proves beneficial for enhancing grasping detection accuracy.

**Figure 6:**
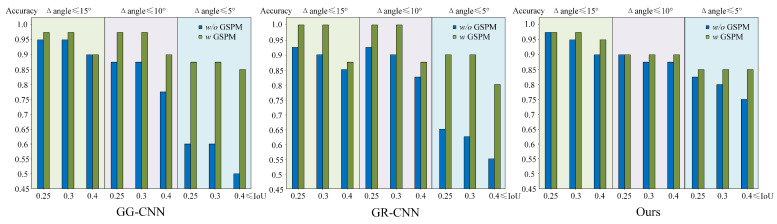
Histogram of grasping detection accuracy based on different methods and evaluation standards. (***w*** **GSPM**) indicates the utilization of both visual and tactile information; ***w*****/*****o*** **GSPM** denotes solely relying on visual information.

As depicted in Figure 6, we observe that as the evaluation standard becomes more stringent, the grasping detection accuracy of our method experiences a decline. However, compared to GG-CNN and GR-CNN, the fluctuations in accuracy are minimal. In particular, when GSPM is not utilized, GG-CNN and GR-CNN exhibit larger fluctuations under strict evaluation standards. Nevertheless, the introduction of GSPM mitigates these fluctuations, leading to a more stabilized performance.

**Figure 7:**
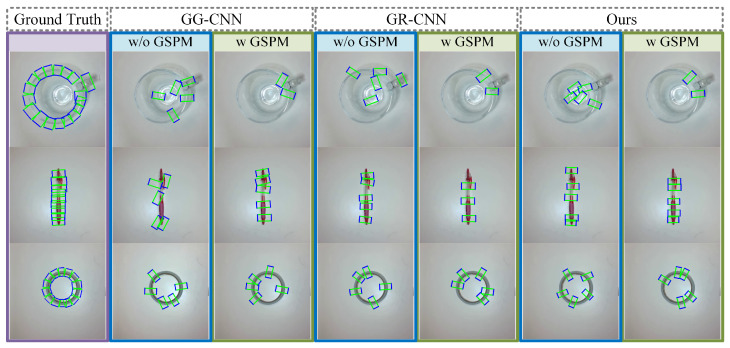
Grasping detection prediction results. The prediction results for grasping detection across various methods reveal a distinct difference when the GSPM is incorporated. In comparison to the ground truth, it is evident that the utilization of the GSPM imposes a clear constraint on the prediction outcomes. This constraint ensures that all grasping rectangles are confined within the grasped region, offering a more precise localization of the object being grasped.

We acquire the prediction outcomes for grasping detection, as illustrated in Figure 7. Upon comparing these findings with the ground truth, it is evident that grasping methods incorporating the GSPM exhibit significantly superior performance compared to those lacking GSPM integration. This improvement stems from the GSPM-based methods’ ability to impose specific constraints on prediction outcomes, introducing a systematic approach that refines the detection process. In doing so, this constraint-imposing method not only enhances prediction accuracy but also furnishes a more interpretable framework for analyzing grasping behavior. Consequently, the integration of the GSPM not only refines but also optimizes the grasping detection process, offering a pathway to enhance overall system performance.

**Figure 8:**
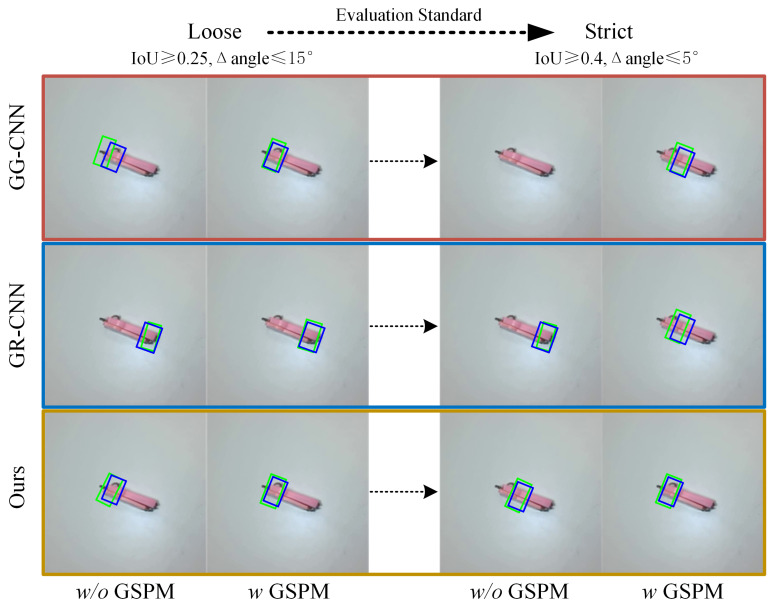
Grasping detection results under varying evaluation standards. This highlights the consistent performance of grasping detection methods integrated with the GSPM. Even amid the transition from a loose to a strict evaluation standard, these methods consistently maintain their efficacy in achieving accurate grasping detection results. The green rectangles denote predicted grasping rectangles, and the blue rectangles are ground truth.

As depicted in Figure 8, we show the grasping detection results for a nail clipper under different methods and present the corresponding ground truth. We observe that even with a shift in evaluation standards from loose to strict, grasping detection methods utilizing GSPM consistently yield favorable results. In the context of robot grasping manipulation, it is noteworthy that while a loose evaluation standard may enhance grasping detection accuracy, it may not necessarily translate to improved performance in robot grasping manipulation tasks.

**Figure 9:**
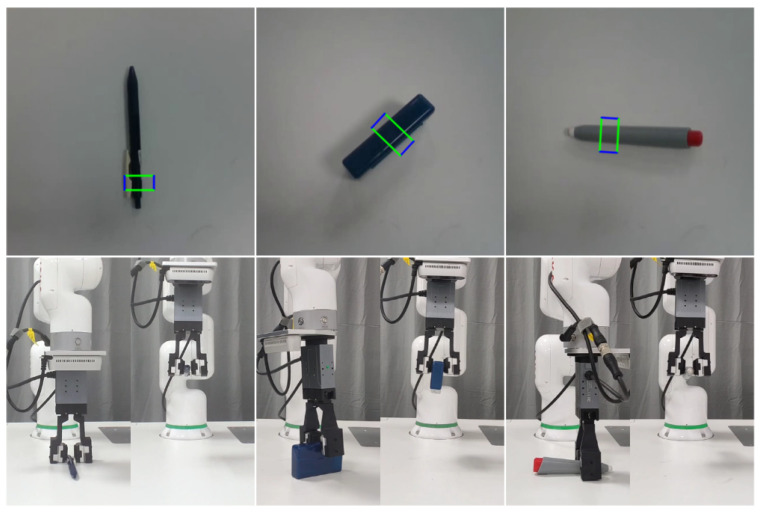
Exemplifying robot grasping of unseen objects in real-world environment using our method. The first row shows the generation of the grasp position by our method. In the second row, the left side shows the robot grasping the object, and the right side shows the robot retracting after a successful grasp. Notably, the objects utilized in this experiment were previously unseen, with their placement and orientation randomized.

We proceeded with evaluating our method on a real robotic platform, as depicted in Figure 9. This showcases various instances of the robot successfully grasping objects. The outcomes of these grasping experiments affirm the capability of our method to accurately predict both the grasping position and rotation angle, even for unseen objects. Ultimately, the gripper jaws successfully grasp the objects.

## 6. Conclusions

This paper introduces a novel robotic grasping detection method that integrates visual and tactile perception. We commence by constructing a comprehensive visual–tactile database, comprising visual object information alongside tactile video sequences capturing potential grasping locations. Subsequently, we introduce the Grasp Stability Prediction Module (GSPM), designed to generate probability maps indicating grasping stability at various object locations. These maps offer valuable prior knowledge to the grasp detection network, enhancing its ability to identify stable grasping positions. Our approach involves feeding the grasp stability probability maps and colored images into the grasp detection network, multiplying them at the pixel level to predict feasible grasping positions. Through extensive experimentation, we evaluate the grasping detection accuracy of methods with and without the GSPM in terms of diverse evaluation criteria. The results demonstrate that our approach, leveraging fused visual and tactile perception, significantly enhances object grasping detection accuracy. In our future endeavors, we plan to expand our visual–tactile database further and delve deeper into the intricate relationships between visual and tactile information.

## Figures and Tables

**Table 1:** Object categories and properties in the visual–tactile grasp dataset. Object size levels are defined as small (<10 cm), medium (10–20 cm), and large (>20 cm) based on the longest dimension.

Object	Category	Material	Size Level
Screwdriver (×3)	Tool	Metal, Plastic	Medium
Hammer	Tool	Metal, Rubber	Large
Tape (×3)	Household	Plastic	Medium
Scissors	Tool	Metal, Plastic	Medium
Pen (×2)	Stationery	Plastic	Medium
Marker	Stationery	Plastic	Medium
Nail cutter	Tool	Metal, Plastic	Small
Glass cup	Container	Glass	Medium
Eyedrop bottle	Container	Plastic	Small
Correction tape	Stationery	Plastic	Medium

**Table 2:** Comparative experiment results based on different methods and evaluation standards.

Method	Evaluation Standard		Accuracy	
	IoU	∆ Angle	*w/o* SPM	*w* GSPM
GG-CNN [14]	0.4	15°	0.900	0.900
		10°	0.775	0.900
		5°	0.500	0.850
	0.3	15°	0.950	0.975
		10°	0.875	0.975
		5°	0.600	0.875
	0.25	15°	0.950	0.975
		10°	0.875	0.975
		5°	0.600	0.875
GR-CNN [35]	0.4	15°	0.850	0.875
		10°	0.825	0.875
		5°	0.550	0.800
	0.3	15°	0.900	1.000
		10°	0.900	1.000
		5°	0.625	0.900
	0.25	15°	0.925	1.000
		10°	0.925	1.000
		5°	0.650	0.900
Ours	0.4	15°	0.900	0.950
		10°	0.875	0.900
		5°	0.750	0.850
	0.3	15°	0.950	0.975
		10°	0.875	0.900
		5°	0.800	0.850
	0.25	15°	0.975	0.975
		10°	0.900	0.900
		5°	0.825	0.850

## Data Availability

The data presented in this study are available upon request from the corresponding author.
